# IL-22 Paucity in APECED Is Associated With Mucosal and Microbial Alterations in Oral Cavity

**DOI:** 10.3389/fimmu.2020.00838

**Published:** 2020-05-08

**Authors:** Epp Kaleviste, Malte Rühlemann, Jaanika Kärner, Liis Haljasmägi, Liina Tserel, Elin Org, Katarina Trebušak Podkrajšek, Tadej Battelino, Corinna Bang, Andre Franke, Pärt Peterson, Kai Kisand

**Affiliations:** ^1^Department of Biomedicine, Institute of Biomedicine and Translational Medicine, University of Tartu, Tartu, Estonia; ^2^Institute of Clinical Molecular Biology, University of Kiel, Kiel, Germany; ^3^Estonian Genome Centre, University of Tartu, Tartu, Estonia; ^4^University Medical Centre Ljubljana, University Children’s Hospital, Ljubljana, Slovenia; ^5^Faculty of Medicine, University of Ljubljana, Ljubljana, Slovenia

**Keywords:** APECED, AIRE, IL-22, oral mucosa, microbiota

## Abstract

Autoimmune polyendocrinopathy-candidiasis-ectodermal dystrophy (APECED) is caused by recessive mutations in the *AIRE* gene. The hallmark of the disease is the production of highly neutralizing autoantibodies against type I interferons and IL-22. Considering the importance of IL-22 in maintaining mucosal barrier integrity and shaping its microbial community, we sought to study potential changes in the oral cavity in this model of human IL-22 paucity. We found that besides known Th22 cell deficiency, APECED patients have significantly fewer circulating MAIT cells with potential IL-22 secreting capacity. Saliva samples from APECED patients revealed local inflammation, the presence of autoantibodies against IFN-α and IL-22, and alterations in the oral microbiota. Moreover, gene expression data of buccal biopsy samples suggested impaired antimicrobial response and cell proliferation, both of which are processes regulated by IL-22. Our data complement the knowledge gained from mouse models and support the concept of IL-22 being a critical homeostatic cytokine in human mucosal sites.

## Introduction

Autoimmune polyendocrinopathy-candidiasis-ectodermal dystrophy syndrome is a rare recessive monogenic disorder (1). The responsible gene, *AIRE*, is expressed in the thymic medullary epithelial cells to support the negative selection of thymocytes that are potentially reactive to peripheral tissue-specific antigens (2–5). Nevertheless, among the autoimmune manifestations, the impaired clearance of *Candida* infection from the mucosal surfaces stands out as the most common and, usually, the first manifestation of the disease (6, 7). It is well established that CMC correlates with circulating autoantibodies against Th17 related cytokines IL-22 and IL-17F (8–10), and that the secretion of the respective cytokines is severely impaired in the circulating and skin-residing CD4+ T cells (9, 11). The potential pathogenicity of the IL-22-neutralizing autoantibodies, isolated from APECED patients, has been confirmed in a mouse model of oropharyngeal candidiasis, where the antibody treatment caused delayed clearance of the yeast from the oral cavity (12).

IL-22 is essential for mucosal barrier function. It can protect from intestinal injury by supporting epithelial cell proliferation and wound healing, enhancing tight junctions, upregulating antimicrobial peptide, and mucus production (13–16). Moreover, IL-22 was recently shown to protect intestinal stem cells against genotoxic stress and thus against colon cancer (17). IL-22 is also capable of shaping gut microbiota (18). In contrast, the excessive production of IL-22 is associated with tissue inflammation in several immune-mediated inflammatory diseases, such as psoriasis, celiac disease, and rheumatoid arthritis (19–23).

While the target cells of IL-22 action are mostly epithelial cells, the best producers of IL-22 are various lymphoid cells: Th17 (24), Th22 (25), type 3 innate lymphoid cells (26), and several unconventional T cells, such as γδ T (27), MAIT (28, 29), NKT (30), and invariant NKT cells (31). Unconventional T cells are crucial for the protection and homeostasis of the epithelial surfaces due to their immediate response to harmful agents. However, they are less studied in APECED patients than are conventional T cells.

Most of the knowledge about the functions of IL-22 have been derived from mouse and *in vitro* experiments. We reasoned that APECED can be viewed as a model disease that enables to study the consequences of IL-22 insufficiency in human oral mucosa. The shortage of IL-22 in APECED is thought to be associated with CMC, but considering the importance of this cytokine for epithelial cell homeostasis in the digestive tract, we hypothesized that it should lead to various other important consequences for epithelial homeostasis.

## Materials and Methods

### Subjects

We studied 13 patients with APECED (9 males, 4 females) from Slovenia and Estonia and 16 control subjects, who were age and gender adjusted for the study, and recruited at the same time with the patients. Use of human material was approved by local ethics committees (Slovenia: National Medical Ethics Committee number 22/09/09 and 28/02/13; Estonia: Research Ethics Committee of the University of Tartu, 235/M-23). Informed consent was obtained from all participants or parents of participating children. Patient details are given in Supplementary Table 1.

### Material

Peripheral blood was drawn into heparinized vacutainers, separated into plasma and peripheral blood mononuclear cells and stored at −20°C or liquid nitrogen, respectively, until usage. The saliva samples were provided using the passive-drool method, in which study participants allowed saliva to pool in the mouth and then drool it into a tube. Donors did not eat or drink for 30 min before sample collection. Samples were stored at −80°C. Buccal biopsies were taken with surgical scalpel under aseptic conditions after local anesthesia. A core from the buccal mucosa of either the left or right cheek was obtained, rinsed in sterile PBS and snap frozen. None of the studied patients or controls received immunosuppressive treatment, reported sicca symptoms nor had difficulties in saliva collection.

### Flow Cytometry

Surface marker expression on PBMCs was assessed by flow cytometry in 8 patients and 8 age matched control subjects. Cells were stained in flow cytometry buffer (PBS (pH 7.2), 2 mM EDTA, 0.5% BSA) for 20 min at room temperature in dark with antibodies listed in Supplementary Table 2. After staining, cells were analyzed using LSRFortessa flow cytometer (BD Biosciences) and FCS Express 5 Flow (*De Novo* Software). The optical detector configuration can be found in Supplementary Table 3. The gating strategy is depicted in Supplementary Figure 1.

### Autoantibodies From Plasma and Saliva With LIPS

Luciferase based immunoprecipitation system assay was conducted on saliva samples (10 APECED patients and 10 controls) and on plasma samples (13 APECED patients and 7 controls). LIPS assay was performed as previously described (32). Briefly, IFN-α, IL-17A, IL-17F, and IL-22 coding sequences were cloned into modified pPK-CMV-F4 fusion vector (PromoCell GmbH, Germany) where Firefly luciferase was substituted with NanoLuc luciferase (Promega, United States). Cloned constructs were transfected into HEK293 cells and secreted luciferase fusion proteins were collected with culture medium after 48 h. IgG from plasma and saliva samples was captured onto Protein G Agarose beads (Exalpha Biologicals, United States) at room temperature for 1 h in 96-well microfilter plate (Merck Millipore, Germany). Next, 1 × 10^6^ luminescence units (LU) of antigen was added per well. After 1 h of incubation the unbound antigen was washed away with vacuum system and Nano-Glo^®^ Luciferase Assay Reagent (Promega, United States) was added. Luminescence intensity was measured by VICTOR X Multilabel Plate Reader (PerkinElmer Life Sciences, United States). The results were expressed as luminescence units (LU) representing the fold over the mean of the heathy control samples.

### Cytokines From Saliva and Plasma

Cytokine quantification was conducted on saliva samples (10 Slovenian patients and 8 controls, with exception for TNF-α, IL-2, IL-1β, IL-5, IL-6, IL-10, GM-CSF, and IFN-γ which were measured with 6 patient samples and 5-6 control samples) and on plasma samples (8 patients and 9 controls, with the exception of TNF-α, IL-2, IL-1β, IL-5, IL-6, IL-10, GM-CSF, and IFN-γ, which were measured from 7 patients and 8 controls). Cytokine levels in saliva samples and EDTA-treated plasma samples were measured by the xMAP Technology on Luminex 200 (Luminex Corp., Austin, TX, United States) with Human Magnetic Luminex Performance Assay Kits (R&D Systems, Minneapolis, MN, United States) (Supplementary Table 4). The cytokine levels were analyzed in accordance with the manufacturer’s protocol. The cytokines studied are listed in Supplementary Table 5 including their lower limits of detection. The values of IL-1α in plasma samples and IL-2, IL-5, IL-17A, IL-17F, and IL-22 in both sample types fell below the detection limits in the majority of tested samples and therefore were excluded from further analysis.

### Gene Expression From Buccal Biopsy

Buccal biopsy samples were derived from 4 patients and 4 age-matched healthy controls. However, after RNA isolation, 2 control samples did not pass the RNA quality control, and therefore, the gene expression analysis was conducted on 4 APECED patient and 2 control buccal biopsy samples. The biopsy specimens were homogenized in TRIzol (Invitrogen) and RNA in the aqueous phase was extracted with the miRNeasy Mini Kit combined with the RNase-free DNase I treatment (both from Qiagen). The concentration and quality of the RNA were assessed with an Agilent RNA 6000 Nano Kit on Agilent 2100 Bioanalyzer (Agilent Technologies, CA, United States). Next, cRNA was prepared from 300 ng of total RNA using an Illumina TotalPrep RNA Amplification Kit (Ambion Inc., TX, United States) according to the manufacturer’s protocol and a genome-wide gene expression analysis was carried out using HumanHT-12 v4 Expression BeadChip (Illumina Inc., CA, United States) and the signals were scanned using a Beadscan (Illumina Inc.). Array data were analyzed using GenomeStudio software (Illumina, San Diego, CA, United States). Rank invariant normalization was applied. Genes with the FDR adjusted *p*-value < 0.05 and a fold change > 1.5 were considered to be differentially expressed. A positive diffScore represents upregulation, while a negative diffScore represents downregulation. The *p*-value for an observed expression difference between two analyzed groups of samples is <0.05 for genes with diffScores of lower than −13 and higher than +13 (*p*-value < 0.01 is −20 > diffScore > 20; *p*-value < 0.001 is −33 > diffScore > 33). The data with negative average signal value was excluded from the list. Annotation analysis was implemented with gprofiler software (33) and IPA software (QIAGEN Inc)^1^. Analysis data set included the upregulated and downregulated gene list with expression fold change. The core analysis was carried out on reference set Ingenuity^®^ Knowledge Base (Genes only). The settings were by default including direct and indirect relationships between genes, 35 genes per network, 25 networks per analysis. Diseases and functions analysis was conducted, which predicted cellular processes and biological functions, based on gene expression. *Z*-score provides predictions about upstream or downstream processes. Network analysis generated the scores [*p*-score = - log10 (*p*-value)] according to the fit of the set of supplied genes and a list of biological functions. Focus Genes were those from the uploaded list that pass filters and are eligible for generating networks. Only genes with a fold change of 1.5 and *p*-value ≤ 0.05 were considered. Upstream regulator analysis in IPA was used to predict the upstream transcriptional regulators from the dataset based on the literature and compiled in the Ingenuity Knowledge Base. Upstream regulator analysis calculated the causal networks. The causal networks scores were calculated using causal paths only. Two *p*-values we calculated: Fishers Exact Test of whether there was a greater than expected proportion of downstream data set genes than expected by chance. Network bias corrected *p*-value was a measure of how often a more significant result was seen in 10K iterations of selecting random data sets of genes with similar relationship number.

### *Candida* Detection From Saliva

DNA from saliva samples and cultured laboratory strain SC5314 (ATCC, VA, United States) of *Candida albicans* was extracted using the DNeasy PowerSoil Kit (QIAGEN) according to manufacturer’s protocol. Samples were homogenized with Precellys 24 Homogenizer (Bertin Instruments) with program 6500 rpm, 2 min’ homogenization and 1-min break. Real time quantitative PCR was performed using Applied Biosystems^®^ ViiA^TM^ 7 Real-Time PCR System with 384-Well Block (Life Technologies) and Maxima SYBR Green/ROX qPCR Master Mix (Fermentas). A primer pair of highly conserved *Candida* rDNA-coding region 28S was used (forward *CGGCGAGTGAAGCGGCTAA*, reverse *ATTCCCAAACAACTCGACTC*) (34). The concentration of *Candida* cells in patient samples was calculated based on the calibration curve constructed according to the Ct values of serially diluted *C. albicans* probes (starting from 2E7 CFU/ml). The normal range of *Candida* concentration was calculated based on healthy control values: mean + 3 standard deviations.

### Sequencing, Processing and Statistical Analysis of Bacterial 16S rDNA Sequences

DNA from saliva samples was manually extracted using the MolYsis^TM^ Complete 5 Kit according to manufacturer’s protocol with 20 μl elution of DNA in water. The bacterial 16S rDNA variable region V1-V2 amplicon libraries were prepared using the 27F-338R primer combination in a dual indexing approach. Sequencing was performed on an Illumina MiSeq device using 2 × 300 cycles and MiSeq Reagent Kit v3 (35). The Dataset included 6 Slovenian patient and 6 control samples. Raw-Data were processed using the DADA2 workflow^2^ (v1.10) and subsequently analyzed in R using the vegan and phyloseq packages. Taxonomic annotation was performed using a Bayesian classifier and RDP training set 16. For each sample, 20k sequences were randomly sampled for a normalized read count. Analysis was performed on ASV, species and genus level. For differentially abundant taxonomic group all tests were performed using Wilcoxon rank sum test using only taxa with median abundance > 100 sequence counts (0.5% rel. abundance) and prevalence > 0.3 (present in at least 4 samples). Alpha diversity (within sample diversity) was analyzed with Wilcoxon rank sum tests. Diversity indices used were as follows: Observed – Observed taxa with abundance > 0, Shannon – Shannon Diversity Index, Chao1 – Chao1 Estimator to estimate observed and unobserved species count. Differences in beta diversity (Whole Community Composition/inter-sample diversity), were assessed using Bray-Curtis similarity and permutational multivariate analysis of variance as implemented in the *adonis*() function of the *vegan* package for R. The sequences have been deposited in the NCBI Sequence Read Archive Database^3^ under accession number PRJNA601650.

### Statistical Analysis

Statistical analysis was performed using the R statistical software^4^ and GraphPad Software (San Diego, CA, United States).

## Results

### APECED Patients Have Decreased MAIT Cell Proportions in Their Circulation

**FIGURE 1:**
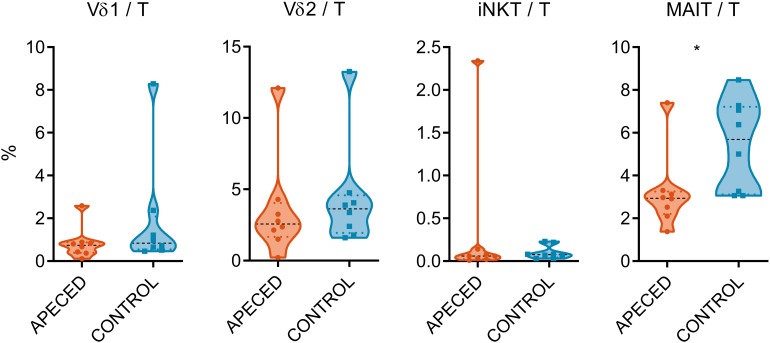
Comparison of circulating lymphocyte subpopulations. Surface marker expression on PBMCs was assessed by flow cytometry in 8 APECED patients and 8 age-matched controls. The percentage of Vδ1+ and Vδ2+ γδ T cells, iNKT and MAIT cells from total T cell pool was compared between patient and control sample groups. In the violin plot the width of distribution of points is proportionate to the number of points at the value of the sample. Black discontinuous line represents median and colored discontinuous line represents quartiles. Statistical significance was assessed with *t*-test using R statistical software (*p < 0.05). The outliers for different cell types did not overlap, with the exception of the APECED patient who had the highest proportion of both γδ T cell subtypes.

With regard to the severe deficiency of Th22 cells in the blood circulation and skin of the APECED patients (9, 11), we enquired if the proportions of unconventional T cells, that are known for their IL-22 production capability, are also impaired in the patients. We used flow cytometry to enumerate these cells (Figure 1) (gating strategy displayed in Supplementary Figure 1). Vδ1+ and Vδ2 + γδ T cell proportions were not significantly altered, in accordance with previous studies (36), neither did the iNKT cell numbers differ between patient and control samples. Nevertheless, the percentages of MAIT cells (Vα7.2 TCR+ and CD161+) among T cells were lower in patients than in healthy controls (*p* < 0.05).

### Saliva From APECED Patients Contain Cytokine Autoantibodies and Reflect Inflammation

**FIGURE 2:**
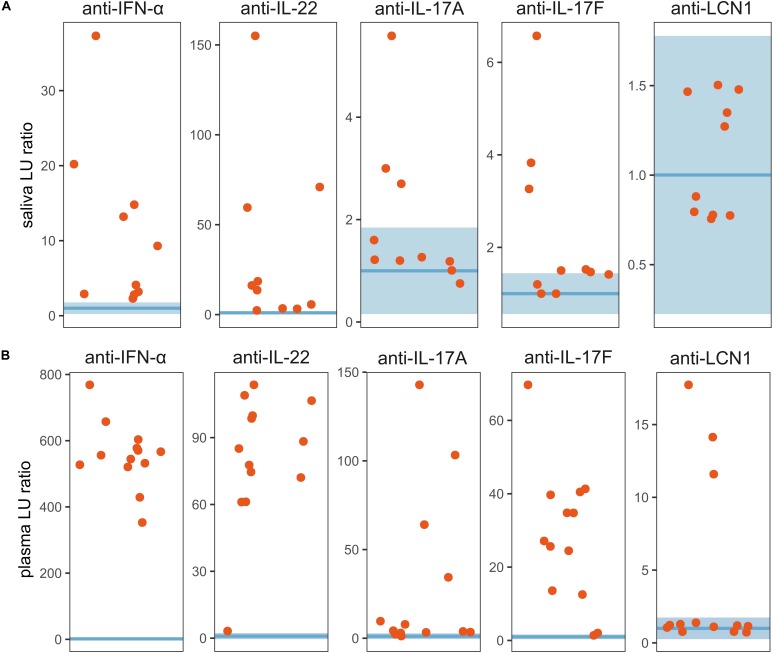
Autoantibodies from saliva and plasma. LIPS assay was conducted on **(A)** saliva samples (10 patients and 10 controls) and on **(B)** plasma samples (13 patients and 7 controls). The results were expressed as luminescence units (LU) representing the fold over the mean of the heathy control samples. The horizontal blue line represents the geometric mean of the LU of the control sample group (saliva *n* = 10, plasma *n* = 7). The transparent blue area shows normal range e.g., ±3 standard deviations of the geometric mean.

As IL-22 and MAIT cells are both important players in mucosal homeostasis and protection, we analyzed saliva samples from patients and controls to find signs of perturbations on oral mucosa. First, we tested the saliva and corresponding plasma samples for the presence of cytokine autoantibodies (Figure 2). Importantly, anti-IFN-α and anti-IL-22 autoantibodies were present in saliva from all the 10 APECED patients. Salivary anti-IL17A and anti-IL17F autoantibodies were detectable at very low levels in a fraction of the patients who tested positive for corresponding circulating autoantibodies. In addition, we detected LCN1 autoantibodies from the plasma of 3 of 13 APECED patients. We also tested for different anti-S100A subtypes (S100A7A, S100A7, S100A8, S100A9) in the plasma, but there was no difference between patient and control groups (Supplementary Figure 2). Collectively, these findings suggest that the local presence of IFN-α and IL-22 autoantibodies can aggravate the insufficiency of these cytokines on mucosal surfaces.

**FIGURE 3:**
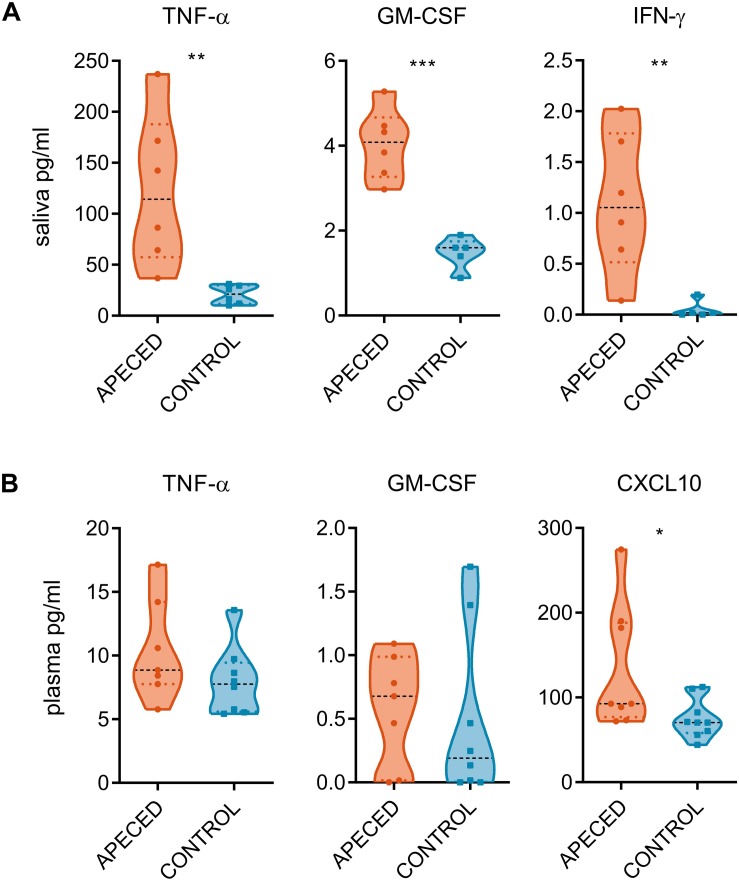
Cytokine quantification. Cytokine measurement assay was conducted on **(A)** saliva samples (6 patients and 5–6 controls) and on **(B)** plasma samples (7 patients and 8–9 controls). Concentration of cytokines from the saliva and from the plasma of patient and control samples groups. In the violin plot the width of distribution of points is proportionate to the number of points at the value of the sample. Black discontinuous line represents median and colored discontinuous line represents quartiles. Statistical significance was assessed with the *t*-test using the Graphpad Prism software (*p < 0.05, **p < 0.01, and ***p < 0.005).

Next, we compared the salivary concentration of various cytokines in APECED patients with those in healthy controls (Supplementary Table 5). IL-22, IL-17A, and IL-17F levels remained undetectable in majority of the samples. However, proinflammatory cytokines TNF-α, GM-CSF, and IFN-γ were all significantly increased compared to control group (Figure 3A). This increase was reflective of the local inflammation as the difference was not recapitulated in the plasma samples (Figure 3B). From tested plasma cytokines, only CXCL10 was significantly increased in patients compared with that in controls, confirming an earlier report (37).

Furthermore, correlation analysis was conducted to compare the *Candida* concentrations (CFU/ml) with significantly increased cytokine levels and autoantibody levels from patient saliva samples (Supplementary Figure 3). There was no correlation between CFU values and proinflammatory cytokine concentrations. The only significant correlation was between salivary anti-IL-17A levels and *Candida* CFU, but this was caused by the one outlier – the patient with the highest *Candida* CFU value had the highest IL-17A antibody level (Supplementary Figure 4).

### Buccal Biopsy Transcriptomes Reveal Alterations in APECED Oral Mucosa

**FIGURE 4:**
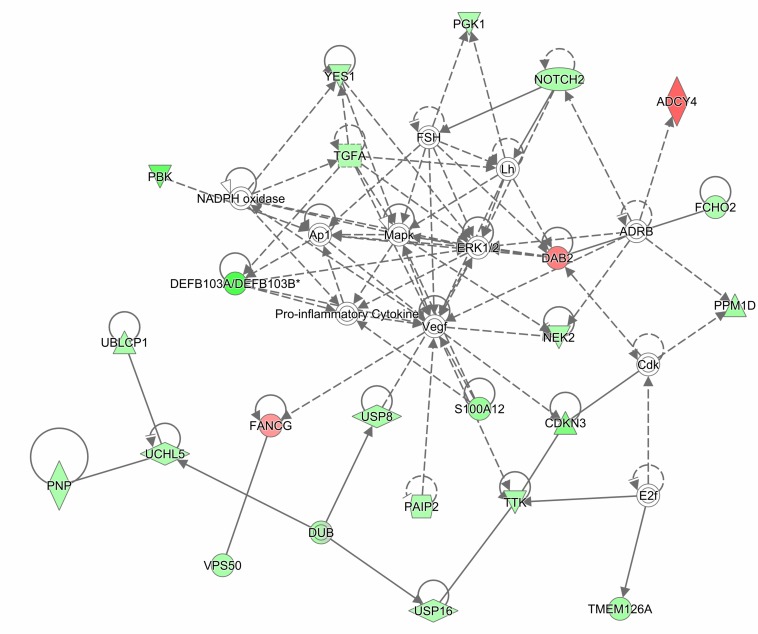
Ingenuity Pathway Analysis (IPA) network diagram of differentially expressed genes in patient vs. control buccal biopsies. The gene expression analysis was conducted on 4 APECED patient and 2 control buccal biopsy samples. Differentially regulated genes were analyzed by IPA. Network analysis with the highest score consisted of 23 focus molecules and 12 interconnecting molecules. Network, which had the highest score (score 51), was categorized by IPA function: “antimicrobial response, cellular function and maintenance, inflammatory response.” Two genes were considered to be connected if there was a path in the network between them. The intensity of the gene color indicates the degree of upregulation (red) or downregulation (green) of a given gene. The legend of figure shapes and relationships can be found in Supplementary Figure 5.

The gene expression analysis was conducted on 4 APECED patient and 2 control buccal biopsy samples. Differentially regulated genes were identified based on gene expression diffScore (Supplementary Table 6). Seventeen significantly up-regulated and 91 significantly down-regulated genes were identified. Among the upregulated genes, several transcripts were associated with tumorigenesis (*SNHG7*, *SHIP2*, *FANCG*, and *DDR2*). In contrast, several AMP genes (*DEFB103B*, *DEFB103A*, and *S100A12*) and genes important for epithelial barrier function (*SPRR2C*, *SPRR2B*, and *TGM5*) were downregulated. The significantly downregulated genes were further analyzed with gProfiler database. Mitotic cell cycle was identified as an associated biological process. In addition, the gene lists together with fold changes were analyzed by IPA. We carried out a diseases and functions analysis, which predicted the biological processes (cellular processes, biological functions, etc.) affected by the expression of a specific gene. The main terms that emerged were connected with cell cycle and mitosis (Supplementary Table 7), the cell cycle process was predicted to be inhibited in patients (*p*-value 3.5E-08, activation *z*-score -2.256). Next, we carried out a network analysis, which predicts gene interaction map (Supplementary Table 8). We focused on the network with the highest score, which consisted of 23 focus molecules and 12 interconnecting molecules (“non-focused” proteins that were not present in our list). The main network included the following biological terms: antimicrobial response, cellular function and maintenance, and inflammatory response (Figure 4 and Supplementary Figure 5).

Finally, we conducted an upstream regulator analysis, which predicts the activity state of regulators by correlating literature reported effects with observed gene expression. Participating regulators are molecules through which the upstream regulator molecule controls the expression of target molecules in dataset. The top two master regulators, identified in the dataset, were *TFIIH* and *AHR*, based on the highest *p*-value of overlap (Supplementary Table 9 and Supplementary Figure 6). Transcription factor IIH (TFIIH) is an important protein complex with many biological roles, ranging from DNA repair to transcription to cell cycle regulation (38). AHR is highly expressed by Th17 cells, and activation of AHR results in expansion of Th17 cells and enhanced production of Th17 cytokines, particularly IL-22 (15, 39, 40).

Taken together, the gene expression array results hint that IL-22-controlled pathways may be downregulated in the buccal biopsy samples of APECED patients.

### Salivary Microbiota Analysis Is Consistent With Dysbiosis in APECED Oral Cavity

**FIGURE 5:**
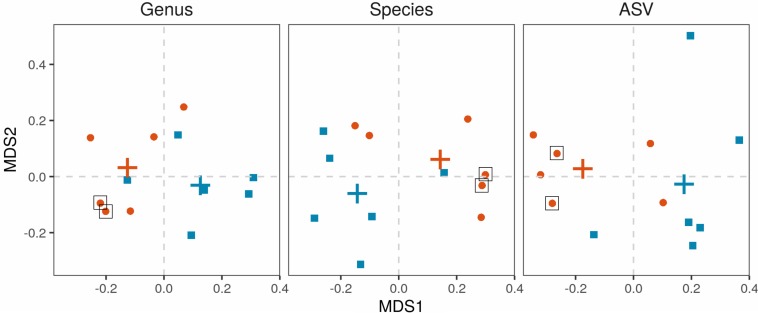
Beta-diversity of the salivary microbiota. Microbiota from salivary samples were analyzed in 6 APECED patients and 6 age-matched healthy controls. Multidimensional scaling based on Bray-Curtis distances was applied with first and second coordinates depicted for genus, species and ASV levels. Each point represents the microbiome of one individual. APECED – orange dots, control individuals – blue squares. Crosses represent centroids per group. Black box – patients with high *Candida* concentration of saliva samples compared to the normal range of *Candida* concentration (healthy control values: mean + 3 standard deviations).

Salivary microbiota was analyzed in 6 APECED patients and 6 age-matched healthy controls. The microbial communities were significantly different in APECED patients in comparison to healthy controls on the whole community (beta-diversity) level (Figure 5). All differences were significant in permutational ANOVA (ASV: *R*^2^ = 14.6%, *p* = 0.017, species: *R*^2^ = 20.2%, *p* = 0.036, genus: *R*^2^ = 23.5%, *p* = 0.021). Interestingly, the 2 patients with elevated *Candida* levels in their saliva samples (Supplementary Table 1 and Supplementary Figure 3), clustered together at the genus and species level (Figure 5).

**FIGURE 6:**
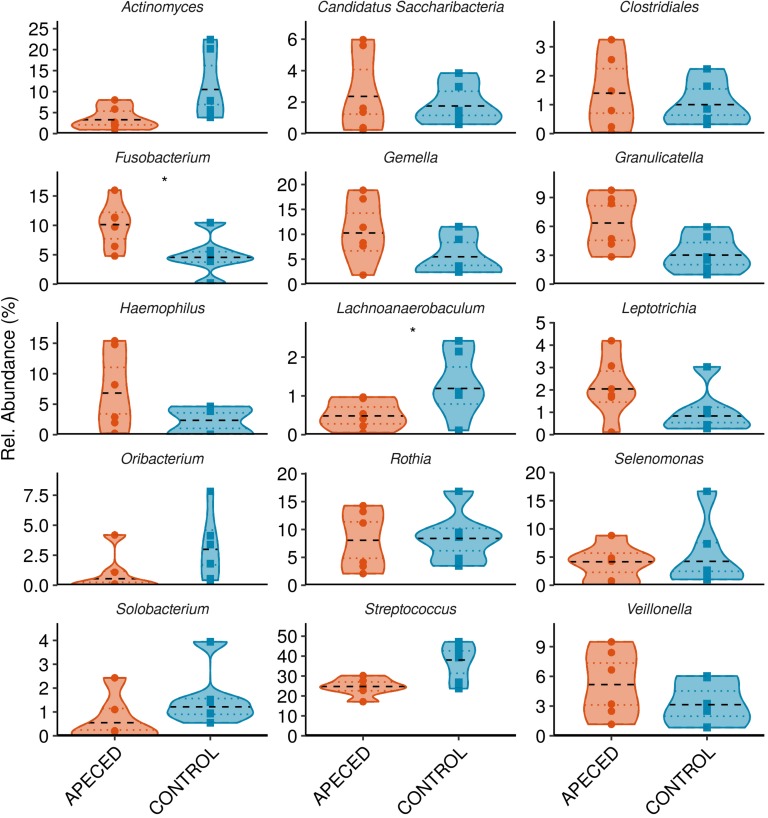
Profiles of the relative abundance per group at genus level in studied groups. Microbiota from salivary samples were analyzed in 6 APECED patients and 6 age-matched healthy controls. Analysis was performed using Wilcoxon rank sum test using only taxa with median abundance > 100 sequence counts (0.5% relative abundance) and prevalence > 0.3 (present in at least 4 samples) (*p < 0.05).

**FIGURE 7:**
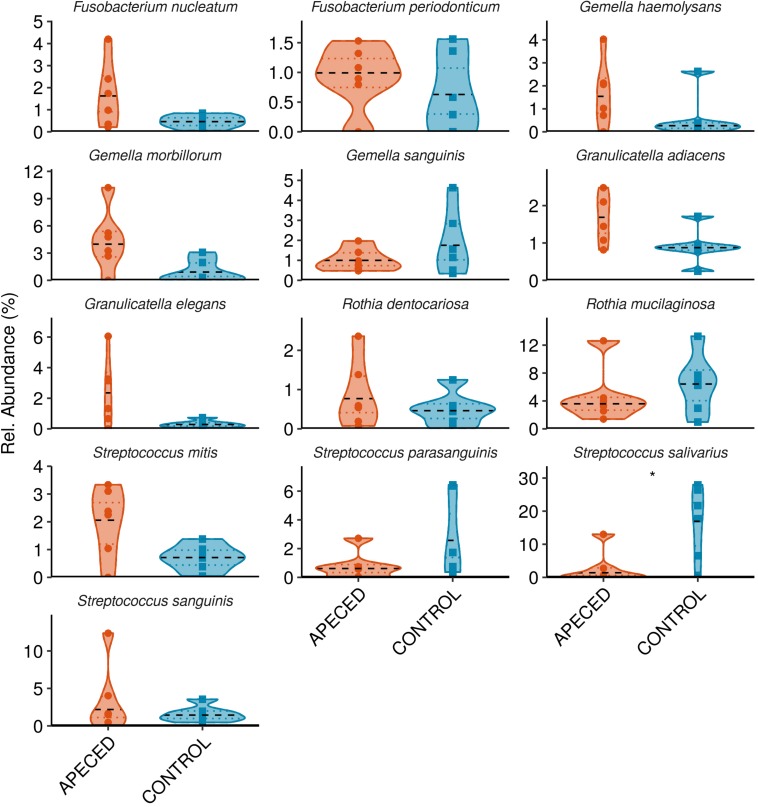
Profiles of the relative abundance per group at species level in studied groups. Microbiota from salivary samples were analyzed in 6 APECED patients and 6 age-matched healthy controls. Analysis was performed using Wilcoxon rank sum test using only taxa with median abundance > 100 sequence counts (0.5% relative abundance) and prevalence > 0.3 (present in at least 4 samples) (*p < 0.05).

Next, we analyzed the taxonomic groups. The most abundant genus in both, patients and controls, was *Streptococcus* (Figure 6). Significantly higher abundance of *Fusobacterium* (*p* < 0.05) was revealed in APECED patients, and a relatively rare genus, *Lachnoanaerobaculum*, was more abundant in heathy individuals. At the species and ASV level, we noted significantly lower abundance of *Streptococcus salivarius* (*p* < 0.05) in APECED patient samples (Figure 7 and Supplementary Figure 7).

**FIGURE 8:**
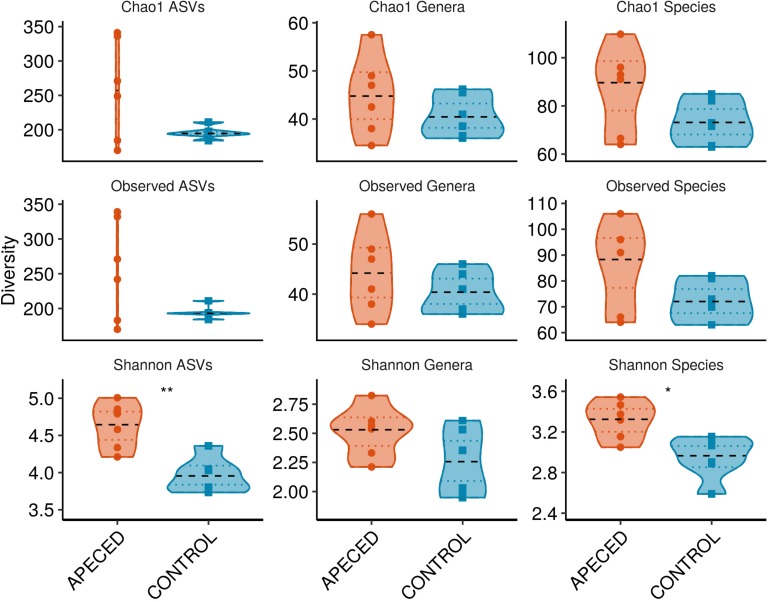
Alpha-diversity analysis of salivary microbiota. Within-sample bacterial diversity was analyzed on ASV, genus and species levels in 6 APECED and respective control samples using three different diversity indices as indicated on the plots (*p < 0.05 and **p < 0.01).

For alpha-diversity analysis, which estimates bacterial diversity within a sample (Figure 8), three different indices were used to estimate counts of observed and unobserved species: observed taxa with abundance over zero, Shannon Diversity Index, and Chao 1 Estimator. Interestingly, significantly increased Shannon diversity was found at the species (*p* < 0.05) and ASV (*p* < 0.01) levels in patient samples. Other alpha-diversity indices showed similar tendencies, but the differences were not significant in this small dataset.

Taken together, the results of the microbial 16S sequencing analysis indicated alterations in oral microbiota of APECED patients and increased alpha-diversity.

## Discussion

Since its discovery almost 20 years ago, IL-22 has gained considerable attention as an essential homeostatic cytokine on epithelial surfaces. IL-22, belonging to Th17 family of cytokines, has similar and even synergistic effects with IL-17A, but it also exerts a plethora of unique effects to secure the barrier function of epithelium (13–16). In this study, we focused on a distinctive feature of APECED patients, namely, highly neutralizing autoantibodies against IL-22 and IFN-α. Previous studies have substantiated that due to neutralization of the low physiologic levels of IFN-α, blood cells of APECED patients have reduced levels of IFN-induced gene expression compared with that of healthy controls (37). Moreover, CMC in APECED patients is associated with the presence of circulating anti-IL-22 and IL-17F (11, 32, 41) and with the incapability of circulating and skin CD4+ T cells to secrete IL-22 after their stimulation *in vitro*. In addition, impaired mucosal barrier function has been suspected in APECED patients due to increased levels of antibodies to commensals (42). In this study, we focused on the events occurring in the human oral mucosa.

The cause for the almost complete absence of IL-22 production by circulating and skin CD4+ T cells in APECED patients is still not known. Furthermore, the role of other lymphoid cells in Th17-related cytokine secreting capacity is not yet clear in this disease. However, there is a previous report suggesting that IL-17A secreting γδ T cells are impaired in APECED (43). According to current study, the percentages of Vδ1+ and Vδ2+ γδ T cells did not differ between patients and controls. The association of iNKT cells with AIRE-deficiency has remained controversial (10, 44, 45). Our study revealed no significant difference between patient and control samples for this cell type. However, we found significantly decreased proportions of circulating MAIT cells in APECED patients. MAIT cells are reportedly present in oral mucosa and are capable of secreting Th17 cytokines (46). Considering this, it is possible that the paucity of MAIT cells contributes to the shortage of IL-22 in mucosal surfaces. The deficiency of MAIT cells in patients can be related to the presence of highly neutralizing autoantibodies against type I IFNs in APECED because MAIT cells are activated by type I IFNs, or by aberrations in microbiota that produce metabolites necessary for MAIT cell development in the thymus (47).

Although the main producers of IL-22 are distinct cell types from the lymphoid lineage (48), which are deficient in APECED patients, dendritic cells, and neutrophils can also contribute to the circulating or local concentration of IL-22 (49, 50). The remaining IL-22 from various sources can be neutralized by IL-22 autoantibodies. Interestingly, we demonstrated autoantibodies against IL-22 and IFN-α also in patients’ saliva samples, levels of which varied a lot between patients. In theory, these local autoantibodies can further aggravate IL-22 shortage although we do not have formal proof for this. One could also hypothesize that local neutralization of IFN-α can further impair the recruitment of cells that could secrete IL-22, as recently reported (51).

As one of the key functions of IL-22 is to induce AMP secretion from epithelial cells in concert with IL-17A, it is possible that the secretion of these natural antibiotics is impaired in APECED patients. However, previous work has not found decreased beta and alpha defensin concentrations in APECED saliva samples (52). Our study confirmed abundant, and not impaired, LCN2 levels in the saliva of APECED patients. Gene expression analysis of buccal biopsy samples revealed equally high S100A8 and S100A9 expression in patients and controls, while some other AMPs (DEFB103B, DEFB103A, and S100A12) were significantly downregulated in the mucosa of APECED patients. These results suggest specific but not universal impairment of AMP production in APECED oral cavity. Interestingly, autoantibodies against antimicrobial peptides, DEFA5 (53), LPO (54), BPIFA1 (54), BPIFA2 (54), and LCN1 (55), have been described previously. In addition, we detected LCN1 autoantibodies in the plasma of three of 13 APECED patients included in this study, but did not detect autoantibodies against the S100A family of antimicrobial peptides.

Considering the importance of the tight regulation of IL-22 levels to enable the symbiosis of the host with commensals (56), we hypothesized that IL-22 paucity could impact microbial community in the oral cavity. Indeed, the salivary microbiome of patients was significantly different from healthy controls (Figure 7) and showed higher diversity. The dysbiosis in APECED patients’ oral cavity has been described in an earlier investigation with some overlaps in differentially abundant taxonomic units between the two studies, but also with discrepancies that can arise from various technical, analytical and biological differences between the two studies (57). Alterations in the microbiota could also be caused by medications, as well as Sjögren’s-like syndrome, which is especially frequent among APECED patients in the United States (10). However, we think that neither factor had impact on our analysis as none of the study subjects received immunosuppressive drugs, and only one patient was on antifungals, all other medications were only hormone replacements. Moreover, objective sicca symptoms are rare in European APECED patients and were reportedly present only in patients over 33 years of age (52). Our study included relatively young patients (17.7 ± 10.9 years) and none of them reported dry mouth. *Candida* infection itself is also a potential modifier of microbiota. According to the beta-diversity analysis (Figure 5) the two patients with elevated *Candida* concentration clustered together at the genus and species level but did not differ from the other patients in any other studied parameter. CMC is also a possible inducer of proinflammatory cytokines that were elevated in the patients’ saliva that in turn can influence the changes in the microbial communities. Although we did not see any significant correlation between *Candida* CFU values and cytokine concentrations, we cannot rule it out by our small study group. The cause of the salivary proinflammatory cytokine increase remains unknown. The oral cavity was regularly examined by the attended pediatric endocrinologist and no clinical sings of gingivitis and periodontitis were appreciated. The autoimmune attack toward mucosal antigens or preclinical inflammation in the salivary glands remains an option.

In addition to shaping the microbiome composition, IL-22 is important for epithelial cell proliferation, enhancing tight junctions and protecting cells from genotoxic stress (17). Indeed, our gene expression data from buccal biopsy samples suggested differences in the upstream and downstream molecular events of the IL-22 pathway. Notably, the upstream regulator analysis identified AHR, the transcription factor necessary for IL-22 production, while network analysis revealed impaired antimicrobial response, cellular function and maintenance, and inflammatory response. The harmed epithelial barrier function can be suspected also from the reduced expression of several genes related with mitotic cell cycle in patients that can lead to defective wound healing on epithelial surfaces that can become especially important during inflammatory processes. Importantly, chronic inflammation due to recalcitrant *Candida* infection has been regarded as a susceptibility factor for developing oral and esophageal squamous cell carcinoma in APECED patients (58). In line with this, the patient saliva samples revealed significantly increased levels of proinflammatory cytokines. As IL-22 was recently shown to protect intestinal stem cells against genotoxic stress and thus against colon cancer (17), we suggest that the susceptibility for oral and esophageal squamous cell carcinoma may result from the combination of persistent inflammation with the lack of protective function of IL-22.

Our study has several limitations including a small sample size and imprecise taxonomic annotation of the 16S amplicons due to the clade-dependent differences in resolution exhibited by the amplicon. Moreover, the usual weakness of the human studies is their descriptive nature and purely indirect reasoning to suggest some causalities.

## Conclusion

According to our results, APECED patients are characterized by alterations in their oral mucosa and increased diversity of their salivary microbiota, both of which can be in theory influenced by IL-22 shortage, possibly aggravated by local action of autoantibodies and paucity of circulating MAIT cells.

## Data Availability Statement

The datasets generated for this study can be found in the NCBI Sequence Read Archive Database, PRJNA601650.

## Ethics Statement

The studies involving human participants were reviewed and approved by National Medical Ethics Committee number 22/09/09 and 28/02/13, Slovenia; Research Ethics Committee of the University of Tartu, 235/M-23, Estonia. Written informed consent to participate in this study was provided by the participant or/and the participants’ legal guardian/next of kin.

## Author Contributions

EK and JK carried out flow cytometry experiments and analyzed the respective data. LH performed LIPS. MR, CB, AF, and EO were responsible for the microbiota analysis. KK, LT, and EK analyzed the gene expression array data. KT and TB recruited the study subjects, collected the samples, and clinical data. KK and PP supervised the research. EK and KK wrote the manuscript with the support of all the other coauthors.

## Conflict of Interest

The authors declare that the research was conducted in the absence of any commercial or financial relationships that could be construed as a potential conflict of interest.

## Footnotes

1 https://www.qiagenbioinformatics.com/products/ingenuity-pathway-analysis/

2 https://benjjneb.github.io/dada2/

3 https://www.ncbi.nlm.nih.gov/sra

4 https://www.r-project.org/

## Abbreviations

AHR: aryl hydrocarbon receptor
AIRE: autoimmune regulator
AMP: antimicrobial peptide
APECED: autoimmune polyendocrinopathy-candidiasis-ectodermal dystrophy
ASV: amplicon sequence variant
CMC: chronic mucocutaneous candidiasis
IPA: ingenuity pathway analysis
LIPS: luciferase based immunoprecipitation system
MAIT: mucosal associated invariant T
NKT: natural killer T
TFIIH: transcription factor IIH.
