# Supplementary material for: IL-22 Paucity in APECED Is Associated With Mucosal and Microbial Alterations in Oral Cavity
Source: Front Immunol. 2020 May 8;11:838. doi: 10.3389/fimmu.2020.00838 (PMC7232598; doi:10.3389/fimmu.2020.00838)
Supplement: Supplementary file 2 [file Data_Sheet_1.pdf]

Supplementary Material

1. Supplementary Figures

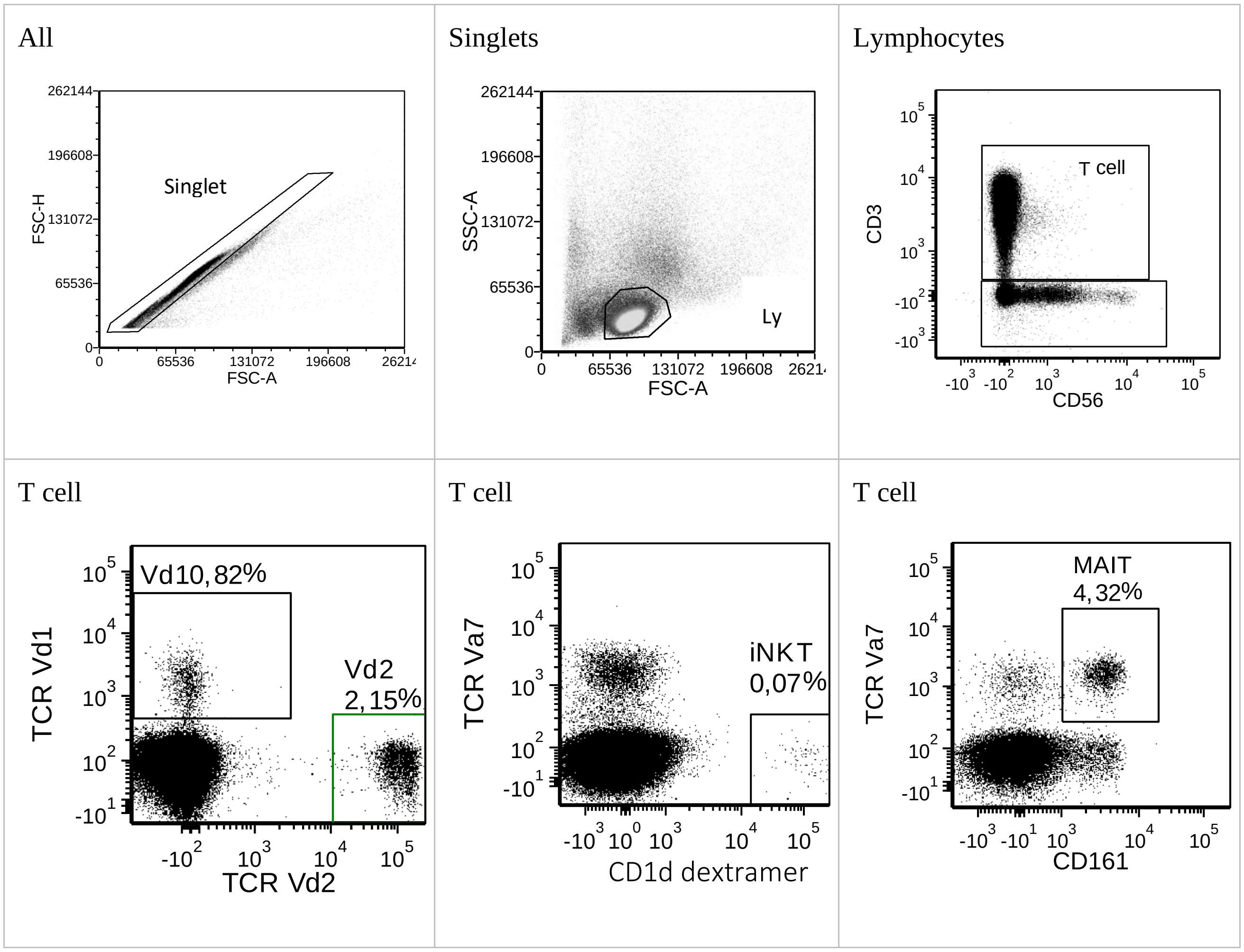

Supplementary Figure 1. Gating Strategy for lymphocyte subpopulations. Surface marker expression on PBMCs was assessed by flow cytometry in 8 Slovenian patients and 8 age matched control subjects. Cells were stained in flow cytometry buffer (PBS (pH 7.2), 2mM EDTA, 0.5% BSA) for 20 min at room temperature in dark with antibodies listed in Supplementary Table 2. After staining cells were analyzed using LSRFortessa flow cytometer (BD Biosciences) and FCS Express 5 Flow (De Novo) software. The optical detector configuration can be found in Supplementary Table 3. Singlets and lymphocytes (ly) are identified by their scatter properties (FSC-A x FSC-H plot, FSC-A x SSC-A plot respectively). Numbers in gates denote percent positive cells.

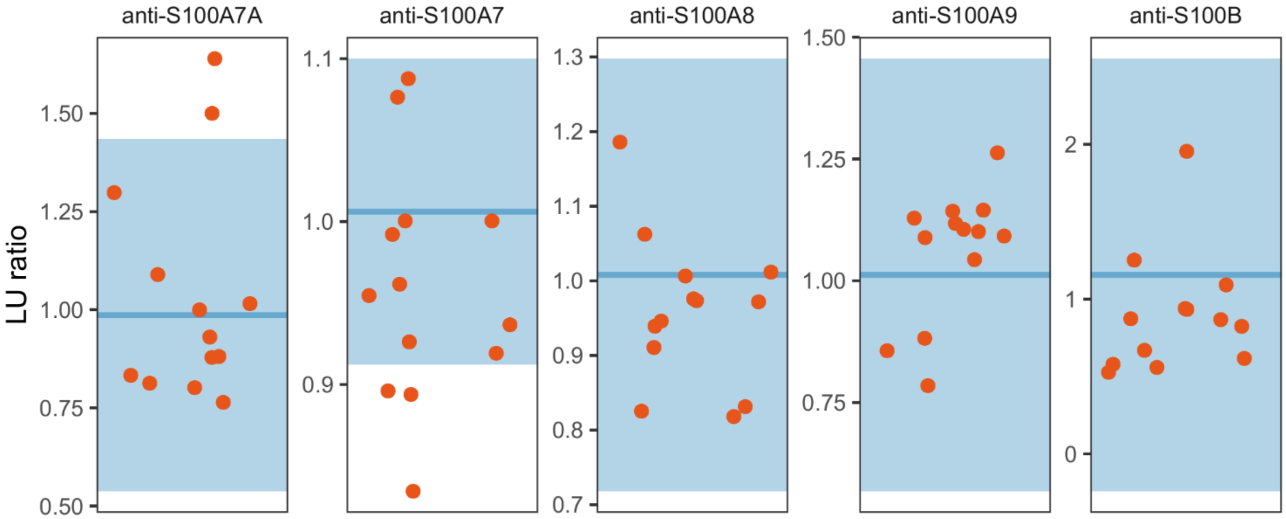

Supplementary Figure 2. Autoantibodies detected from plasma. Luciferase based immunoprecipitation system (LIPS) assay was conducted on plasma samples (13 patients and 7 controls). The results were expressed as luminescence units (LU) representing the fold over the mean of the heathy control samples. The horizontal blue line represents the geometric mean of the LU of the control sample group (plasma n = 7). The transparent blue area shows normal range e.g. ± 3 standard deviations of the geometric mean.

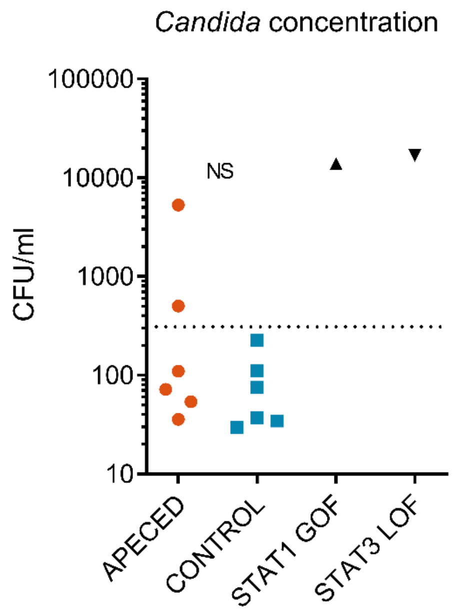

Supplementary Figure 3. *Candida* concentration (CFU/ml) in saliva was measured in 6 APECED patient, 6 control and as a positive control in two patients with different monogenic diseases with CMC: STAT1 GOF and STAT3 LOF. DNA from saliva samples and cultured laboratory strain SC5314 (ATCC, VA, US) of *Candida albicans* was extracted from saliva samples using the DNeasy PowerSoil Kit (QIAGEN) according to manufacturer's protocol and homogenized with Precellys 24 Homogenizer (Bertin Instruments). The concentration of *Candida* cells in patient samples was calculated based on the calibration curve constructed according to the Ct values of serially diluted *C. albicans* probes (starting from 2E7 CFU/ml). The normal range of *Candida* concentration was calculated based on healthy control values: mean + 3 standard deviations (dotted line). Statistical significance was assessed with the unpaired t-test using the Graphpad Prism software. NS – not significant.

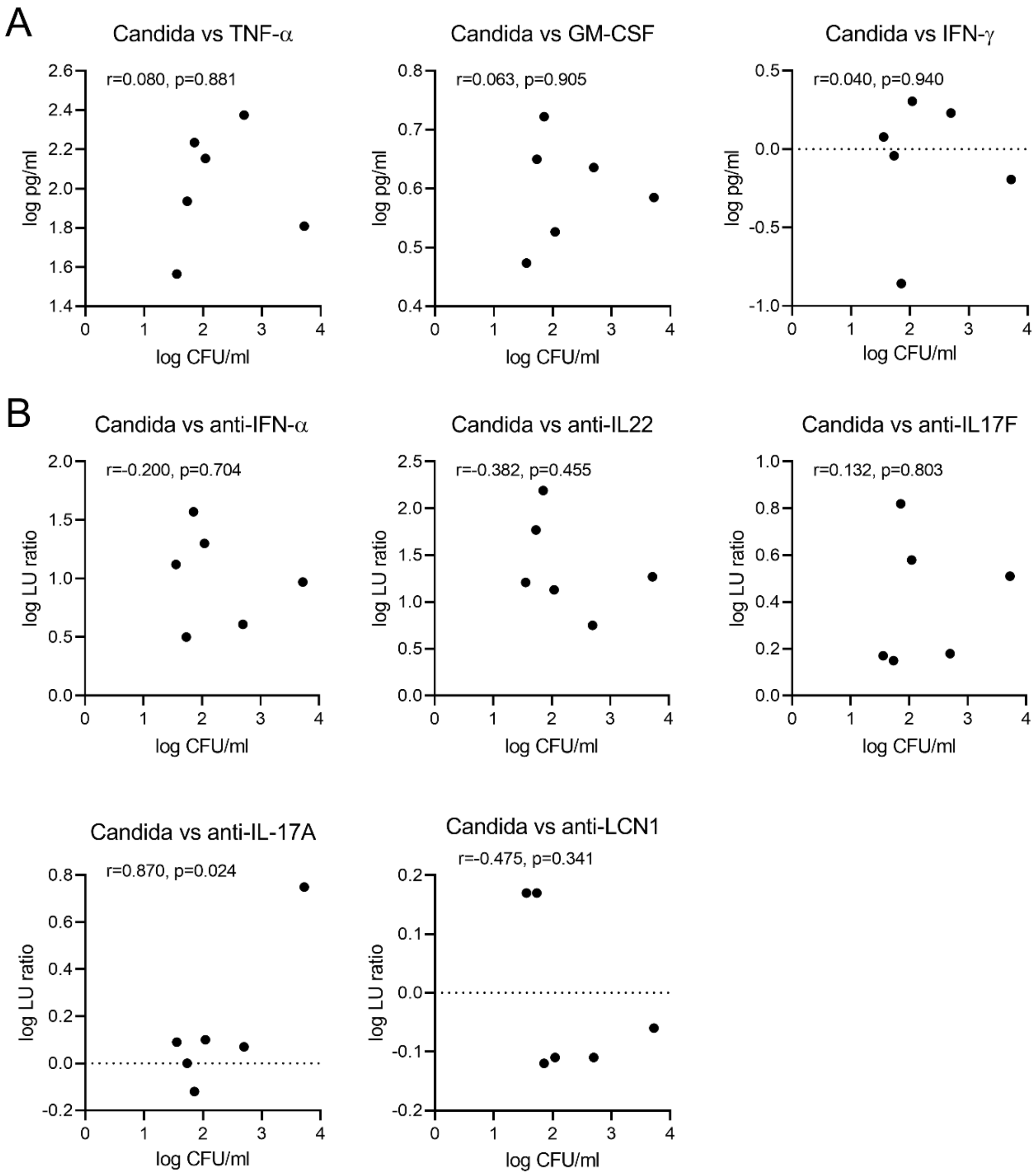

Supplementary Figure 4. Correlation analysis between *Candida* concentration (CFU/ml) and significantly increased cytokines (A) and also autoantibody levels (B) from saliva. Measurements were done in 6 APECED patient samples from saliva. Values were log transformed.

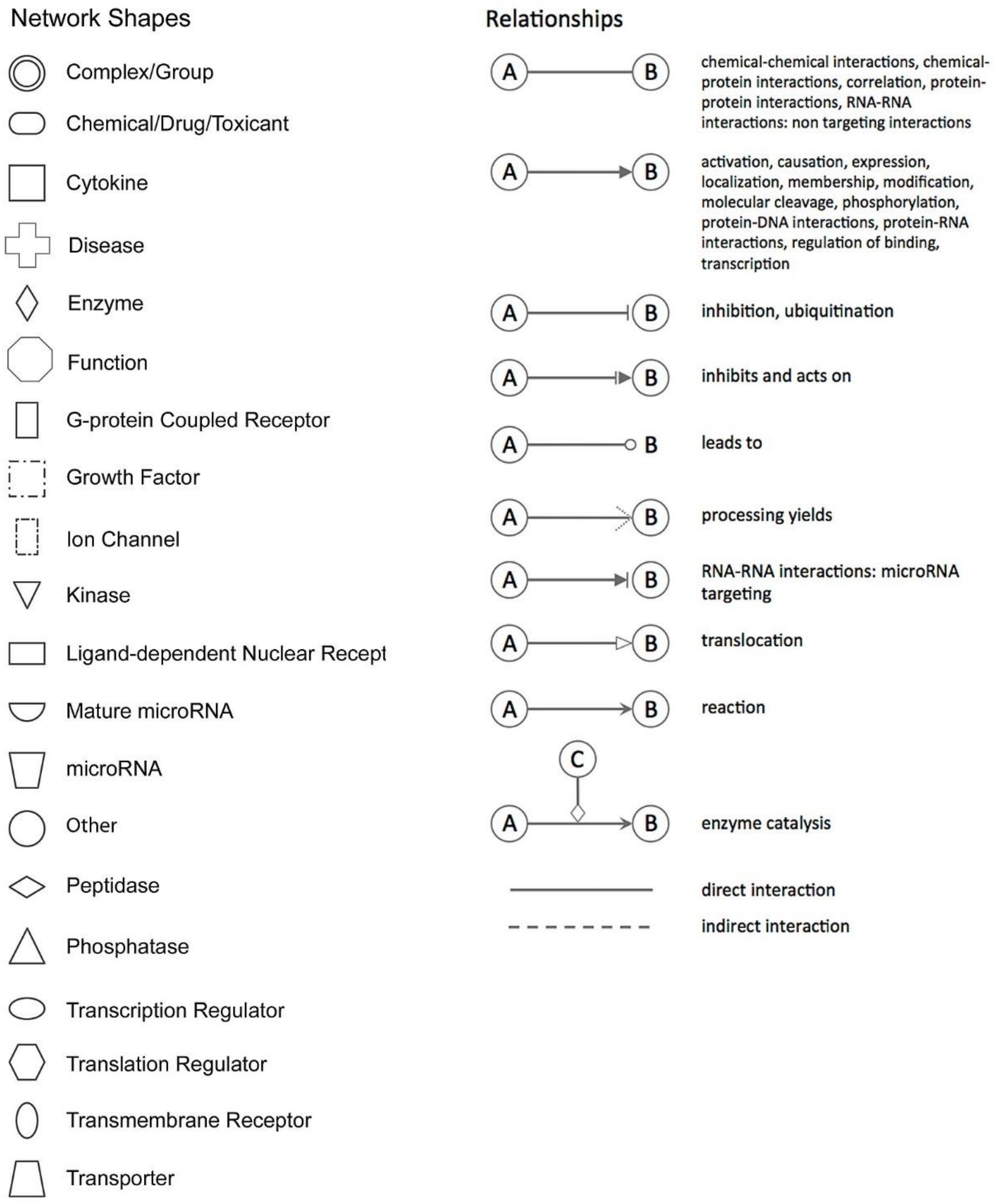

Supplementary Figure 5. Figure legend for the main features of IPA network analysis of Figure 4 in the main text.

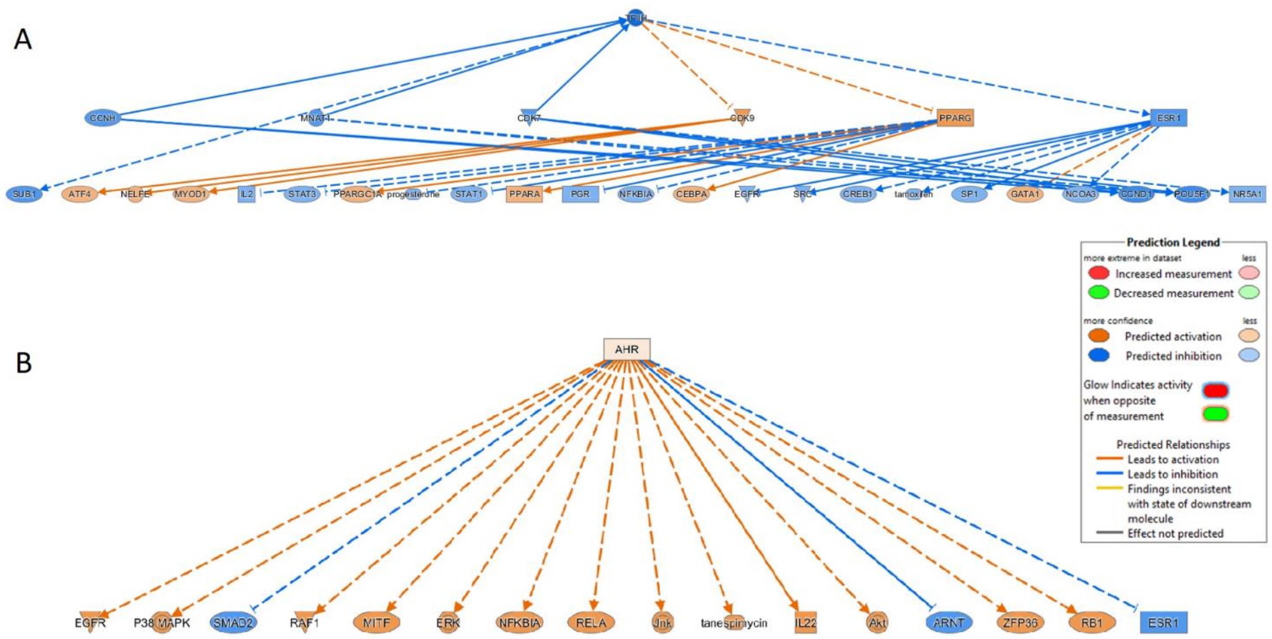

Supplementary Figure 6. Causal networks of top 2 master regulators: TFIIH (A) and AHR (B). The visual representation of the regulatory network that represents possible causes and underlying mechanisms for observed gene expression changes in a given data set. They are constructed hierarchically with a master regulator at the top.

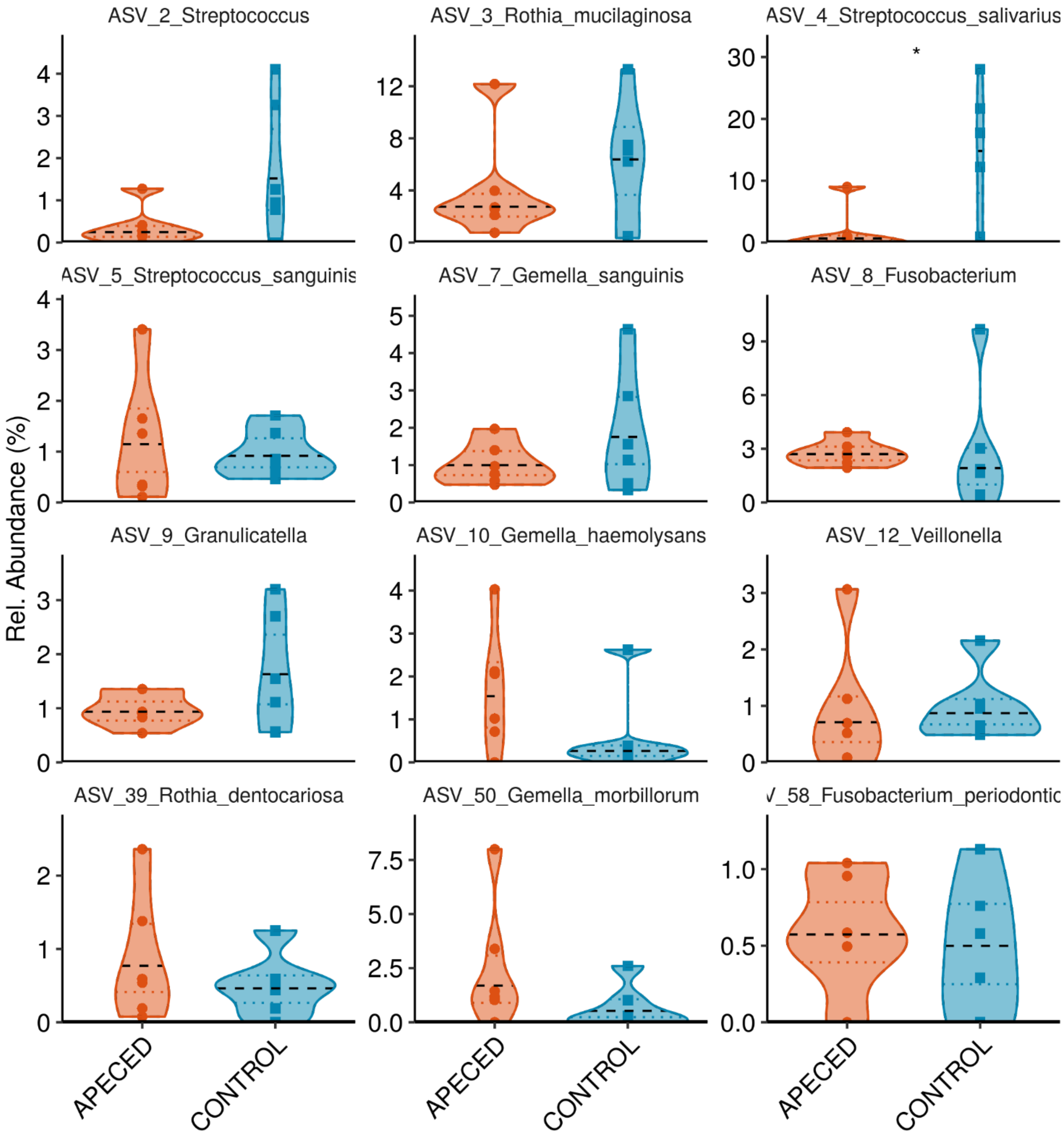

Supplementary Figure 7. Profiles of the relative abundance per group at ASV level in studied groups. Microbiota from salivary samples were analyzed in 6 APECED patients and 6 age-matched healthy controls. Analysis was performed using Wilcoxon rank sum test using only taxa with median abundance > 100 sequence counts (= 0.5% relative abundance) and prevalence > 0.3 (= present in at least 4 samples) (* $p < 0.05$).

2. Supplementary Tables

Supplementary Table 1. Patient details. Abbreviation: CMC - candidiasis, AD - Addison's disease, HP - hypoparathyroidism, GI - gonadal insufficiency, T1D - type 1 diabetes mellitus, GHD - growth hormone deficiency, HT - hypothyroidism, PA - pernicious anemia, AIH - autoimmune hepatitis, MA - malabsorption, GID - gastrointestinal dysfunction, D - diarrhea, O - obstipation, TIN - tubulointerstitial nephritis / kidney disorders related to APECED, K - keratitis/keratopathy, EH - enamel hypoplasia, VIT - vitiligo, AL - alopecia, ND - nail dystrophy, AS - asplenia, PV - pithyriasis versicolor, E - epilepsy 16 years 10 months, AC - allergy to carbamazepine, HC - hypercholesterolemia, HTE - hypertension, DY - dysphagia, STD - staphylodermia, VA - varicella (10 years), SS - short stature, DP - delayed puberty, SD - steroid diabetes (10 years, while he had varicella), OB - obesity, GLI - glucose intolerance, IR - insulin resistance, AN - acanthosis nigricans, SD/AIH - steroid diabetes (16 years 2 months) during relapse of autoimmune hepatitis, B - bronchectasias; OS - osteoporosis. IFNA cocktail included IFNA1, IFNA2, IFNA8, IFNA21. *Candida* was measured from saliva (CFU per ml), the patient samples which exceeded the normal range (healthy control values: mean + 3 standard deviations) are marked in bold.

**Case**	**Mutation**	**Age at sample collection**	**Gender**	**Clinical phenotype**	**Plasma autoantibodies**				***Candida* detection**			**Experiments**			
					**Anti-IFNα coctail**	**Anti- IL-17A**	**Anti- IL-17F**	**Anti- IL-22**	**Visible *Candida* infection in the mouth at the time of the sampling**	**Mouth swap / *Candida* CFU per ml**	**Antifungal treatment**	**Saliva autoantibodies**	**Flow cytometry**	**Expression analysis**	**Microbiota analysis**

**PT1**	R257X/T16M	21	F	CMC, HP, MA, EH	+	-	-	+	no changes visible on mucous membranes	*Candida albicans* positive	no	✓	✓	✓	
**PT2**	R257X/T16M	16	M	CMC, AD, HP, HT, MA, EH, AL	+	-	+	+	no changes visible on mucous membranes	*Candida albicans* neg	no	✓		✓	
**PT3**	21-43dup23bp x2	15	M	CMC	+	+	+	+	no changes visible on mucous membranes	*Candida albicans* neg	no	✓		✓	
**PT4**	1064-1068dupCCCGG x2	23	M	CMC, HP, GI, HT, MA, EH	+	+	+	+	Numerous *Candida* plaques in the mouth, redness of the mucosa	*Candida albicans* positive	no	✓		✓	
**PT5**	R257X/R257X	26	M	CMC, AD, HP, HT, K	+	+	+	+	*Candida* plaques on the tongue	*Candida albicans* positive / **5281**	no	✓	✓		✓
**PT6**	R257X/T16M	23	M	CMC, AD, GI, GHD, HT, PF, K, EH, ND, DD	+	+	+	+				✓	✓		
**PT7**	R257X/R257X	33	M	CMC, AD, HP, MA, V, AL, PV, E, AC, HC, HTE, DY	+	-	+	+	no changes visible on mucous membranes	*Candida albicans* positive / **501**	no	✓	✓		✓
**PT8**	R257X/R257X	20	M	CMC, AD, GI, AIH, AL, STD,	+	+	+	+	the plaques of trush visible on tongue, buccal	*Candida albicans* positive / 110	Lamisil (terbinafine) 1 tbl a 250 mg/ day for 3 months,	✓	✓		✓

				VA, SS, DP, SD, OB, GLI, IR, AN, SD/AIH					mucosa and palate		Mikonazol gel				
**PT9**	21-43dup23bp	8	M	CMC, HP	+	+	+	+	angular cheilitis	minimal *Candida albicans* / 72	no	✓	✓		✓
**PT10**	R257X/R257X	4	F	CMC, HP, EH	+	-	+	+	normal mucous membranes	*Candida albicans* positive / 54	no	✓	✓		✓
**PT11**	R257X/653-7_-5delCTC	15	F	CMC, AD, HP, GI, GHD, K	+	+	+	+	no changes visible on mucous membranes	*Candida albicans* neg / 36	no	✓	✓		✓
**PT12**	R92W/R257X	55	F	HP, AD	+	-	-	+					✓		
**PT13**	R257X/R257X	12	M	CMC, AD, HP, ND	+	-	+	+					✓		

Supplementary Table 2. Used antibodies. Surface marker expression on PBMCs was assessed by flow cytometry in 8 Slovenian patients and 8 age matched control subjects. Cells were stained in flow cytometry buffer (PBS (pH 7.2), 2mM EDTA, 0.5% BSA) for 20 min at room temperature in dark with antibodies.

Antibody and dye	Manufacturer	Clone
APC-Cy7 CD56	Biolegend	HCD56
Brilliant Violet 650 CD3	Biolegend	OKT3
FITC TCR Vd1	Thermo	TS8.2
PE TCR Vd2	Biolegend	B6
APC CD1d dextramer	Immudex	-
Brilliant Violet 510 TCR va7	Biolegend	3C10
PerCP-Cy5.5 CD161	Biolegend	HP-3G10

Supplementary Table 3. Optical detector configuration details. Surface marker expression on PBMCs was assessed by flow cytometry in 8 Slovenian patients and 8 age matched control subjects. After staining cells were analyzed using LSRFortessa flow cytometer (BD Biosciences).

Laser	PMT	Channel	LP Mirror	BP Filter
488-nm blue laser<br>Octagon	A	FL3	685	710/50
	B	FL2	505	530/30
	C	FL1	-	488/10
405-nm violet laser<br>Octagon	A	FL9	670	710/40
	B	FL8	630	670/30
	C	FL7	600	610/20
	D	FL6	535	540/30
	E	FL5	505	525/50
	F	FL4	-	440/40
561-nm YG laser<br>Octagon	A	FL19	750	780/60
	B	FL18	685	710/50
	C	FL17	635	670/30
	D	FL16	600	610/20
	E	FL15	-	586/15
355-nm UV laser	A	FL11	635	740/40

Trigon	B	FL10	-	450/50
640-nm red laser Trigon	A	FL14	750	780/60
	B	FL13	690	730/45
	C	FL12	-	670/14

Supplementary Table 4. Human Magnetic Luminex Performance Assay Kits. Cytokine levels in saliva samples and EDTA-treated plasma samples patients were measured by the xMAP Technology on Luminex 200 with Human Magnetic Luminex Performance Assay Kits (R&D Systems, Minneapolis, MN).

Kits	Cytokines
Human Magnetic Luminex Performance Assay Base kit A	IL-1ra/IL-1F3, IL-1α/IL-1F1, G-CSF, CXCL8/IL8
Human Mag Luminex Performance Assay Base Kit, HS Cytokine A	TNF-α, IL-10, IL-6, IL-5, IL-2, IL-1β/IL-1F2, IFN-γ, GM-CSF
Human Mag Luminex Performance Assay Base Kit, HS Cytokine B	IL-22, IL-17A, IL-17F, IL-36β, IL-7, IL-31
Human Mag Luminex Performance Assay Base kit, Kidney Biomark	Lipocalin-2/NGAL, CXCL10/IP10

Supplementary Table 5. Cytokines detected from saliva and plasma. In plasma samples the measurements of IL-1a fell below detection limit, therefore were excluded from the results. In addition, IL-17A, IL-17F and IL-22 fell below detection limit in both plasma and saliva samples, therefore they were excluded from the results. Lower limit of detection (LOD) according to the manufacturer.

Cytokine	LOD	Sample	Patient n/ Control n	Patient mean ± SD	Control mean ± SD
IL-36β	0.49	Saliva	10 / 8	350.8 ± 232.6	632.9 ± 749.9
		Plasma	8 / 9	3.54 ± 6.87	4.73 ± 10.24
IL-7	0.80	Saliva	10 / 8	9.26 ± 3.70	9.63 ± 8.54
		Plasma	8 / 9	20.36 ±10.29	19.25 ± 14.89
TNF-α	0.81	Saliva	6 / 6	123.0 ± 74.79	20.85 ± 9.17
		Plasma	7 / 8	10.39 ± 3.97	8.02 ± 2.73
IL-1β	0.34	Saliva	6 / 6	452.7 ± 405.0	118.9 ± 120.2
		Plasma	7 / 8	1.13 ± 0.40	1.01 ± 0.57
IL-6	0.93	Saliva	6 / 6	16.50 ± 6.82	12.77 ± 14.79
		Plasma	7 / 8	1.70 ± 0.37	1.74 ± 1.54

IL-10	0.46	Saliva	6 / 5	2.49 ± 2.90	1.65 ± 1.78
		Plasma	7 / 8	5.47 ± 11.40	0.67 ± 0.55
GM-CSF	0.39	Saliva	6 / 5	4.04 ± 0.83	1.47 ± 0.37
		Plasma	7 / 8	0.57 ± 0.44	0.49 ± 0.67
IFN-γ	0.31	Saliva	6 / 5	1.10 ± 0.69	0.04 ± 0.08
		Plasma	7 / 8	0.54 ± 1.25	0.07 ± 0.06
CXCL10/IP10	3.65	Saliva	10 / 8	398.7 ± 345.4	168.9 ± 94.07
		Plasma	8 / 9	133.2 ± 73.92	75.24 ± 23.16
Lipocalin-2/NGAL	41.15	Saliva	10 / 8	99355 ± 26968	119377 ± 27764
		Plasma	8 / 9	33938 ± 22667	32814 ± 18470
G-CSF	5.83	Saliva	10 / 8	72.96 ± 44.06	96.21 ± 37.96
		Plasma	8 / 9	22.01 ± 11.32	20.32 ± 8.52
IL-1α /IL-1F1	2.88	Saliva	10 / 8	349.4 ± 196.0	572.7 ± 540.8
		Plasma	-	-	-
IL-1ra/IL-1F3	6.52	Saliva	10 / 8	64908 ± 20442	73890 ± 34548
		Plasma	8 / 9	1123 ± 856.8	1645 ± 548.3
CXCL8/IL-8	3.77	Saliva	10 / 8	3255 ± 3023	1727 ± 1211
		Plasma	8 / 9	14.31 ± 7.86	12.53 ± 16.10

Supplementary Table 6. Genome-wide gene expression analysis was carried out using HumanHT-12 v4 Expression BeadChip (Illumina Inc., CA, USA) and the signals were scanned using a Beadscan (Illumina Inc.). Array data were analyzed using GenomeStudio software (Illumina, San Diego, CA, USA). Rank invariant normalization was applied. Genes with the FDR adjusted p-value < 0.1 and a fold change >1.5 were considered to be differentially expressed. A positive diffScore represents upregulation, while a negative diffScore represents downregulation. The p-value for an observed expression difference between two analyzed groups of samples is <0.05 for genes with diffScores of lower than -13 and higher than +13 (p-value < 0.01 is -20 > diffScore > 20; p-value < 0.001 is -33 > diffScore > 33).

The Supplementary Table 6 can be found as a separate excel file (the p-values < 0.05 are marked in bold).

Supplementary Table 7. Diseases and functions top 10. The core analysis was carried out on reference set Ingenuity Knowledge Base (Genes only). The settings were by default including direct and indirect relationships between genes, 35 genes per network, 25 networks per analysis. Diseases and functions

analysis was conducted, which predicted effected biology (cellular processes, biological functions) based on gene expression. *P*-value was calculated using a Right-Tailed Fisher's Exact Test.

**Categories**	**Function**	**Diseases or function annotations**	***p*-value**	**Predicted activation state**	**Activation z-score**	**Molecules**	**# molecules**
Cell cycle	Cell cycle progression	Cell cycle progression	3.57E-08	Decreased	-2.256	ACVRL1↑, DDR2 ↑, HAT1 ↓, NEK2 ↓, PPM1D ↓, TTK ↓, CDKN3 ↓, DLGAP5* ↓, HMGB1 ↓, NET1 ↓, RPA3 ↓, UCHL5 ↓, CEACAM1 ↓, DTL ↓, KIF11 ↓, NOTCH2 ↓, SPRR2B ↓, USP16 ↓, CLTC ↓, FANCG ↑, KLF5 ↓, PCLAF ↓, TGFA ↓	23
Cellular function and maintenance	Endocytosis	Endocytosis by cervical cancer cell lines	6.23E-06		-0.628	CLTC ↓, TGFA ↓, FCHO2 ↓, DAB2 ↑, SHANK3* ↑	5
Cell cycle	Interphase	Interphase	7.19E-06		-1.633	ACVRL1 ↑, KIF11 ↓, ORC3 ↓, SNHG7* ↑, CDKN3 ↓, NEK2 ↓, PCLAF ↓, TGFA ↓, DAB2 ↑, NET1 ↓, PPM1D ↓, TNFRSF14 ↑, DTL ↓, NOTCH2 ↓, RPA3 ↓, USP8 ↓	16
Cell cycle	Mitosis	Mitosis	1.78E-05		-0.876	CDKN3 ↓, HAT1 ↓, RPA3 ↓, CEACAM1 ↓, KIF11 ↓, TGFA ↓, CLTC ↓, NEK2 ↓, TTK ↓, DLGAP5* ↓, PPM1D ↓, USP16 ↓	12

Cancer, organismal injury and abnormalities	Breast or colorectal cancer	Breast or colorectal cancer	2.25E-05			ACVRL1 ↑, C1QRNF5 ↑, CLPX ↓, DLGAP5* ↓, FANCG ↑, INPPL1 ↑, METTL5 ↓, NNT ↓, PAK1IP1 ↓, PGK1 ↓, RAD51AP1 ↓, SMPDL3A ↓, TMEM 126A ↓, USP16 ↓, ADCY4 ↑, CD58 ↓, CRYZ ↓, DST ↓, FCHO2 ↓, KIF11 ↓, NEK2 ↓, NOTCH2 ↓, PBK ↓, PLCH2 ↑, SERPING1 ↑, SRP19 ↓, TTK ↓, VSIG8 ↓, AFTPH ↓, CDKN3 ↓, DAB2 ↑, DTL ↓, HAT1 ↓, KLF5 ↓, NET1 ↓, NUP54 ↓, PCLAF ↓, PPM1D ↓, SHANK3* ↑, TAF1B ↓, TXNDC9 ↓, YES1 ↓, BCL10 ↓, CEACAM1 ↓, DDR2 ↑, EIF1AX ↓, HMGB1 ↓, KTN1 ↓, NISCH ↑, OLFML3 ↑, PDCD10 ↓, PRDM1 ↓, SMC2 ↓, TGFA ↓, UCHL5 ↓	55
Cellular assembly and organization, cellular function and maintenance	Formation	Formation of artificial clathrin cages	3.18E-05			CLTC ↓, FCHO2 ↓, DAB2 ↑	3
Cell cycle	Mitosis	Mitosis of tumor cell lines	6.52E-05			CLTC ↓, PPM1D ↓, TTK ↓, DLGAP5* ↓, KIF11 ↓, NEK2 ↓	6

Cell cycle	Mitosis	Mitosis of cervical cancer cell lines	1.13E-04			CLTC ↓, TTK ↓, DLGAP5* ↓, KIF11 ↓, NEK2 ↓	5
Cell cycle	Interphase	Interphase of tumor cell lines	1.16E-04		-1.342	CDKN3 ↓, NET1 ↓, SNHG7* ↑, DAB2 ↑, NOTCH2 ↓, TGFA ↓, DTL ↓, ORC3 ↓, KIF11 ↓, PCLAF ↓	10
Hematological system development and function, hematopoiesis, tissue morphology	Quantity	Quantity of short-term hematopoietic stem cells	1.52E-04			KLF5 ↓, PCLAF ↓	2

Supplementary Table 8. Networks of gene interactions. Networks are assembled based on gene connectivity with other genes using decreasingly connected molecules from data set. The score (p-score = − log10 (*p*-value)) is generated according to the fit of the set of supplied genes and a list of biological functions stored in the Ingenuity Knowledge Base. Focus Genes (or Focus Molecules) are those from the uploaded list that pass filters and are eligible for generating networks, i.e., potentially could be linked to some other genes.

ID	Molecules in network (max 35)	Score	Focus molecules	Top diseases and functions
1	ADCY4 ↑, CDKN3 ↓, NEK2 ↓, PGK1 ↓, S100A12 ↓, UBLCP1 ↓, DAB2 ↑, NOTCH2 ↓, PNP ↓, TGFA ↓, UCHL5 ↓, VPS50 ↓, DEFB103A/DEFB103b ↓, FANCG ↑, PAIP2 ↓, PPM1D ↓, TMEM126A ↓, USP16 ↓, YES1 ↓, FCHO2 ↓, PBK ↓, TTK ↓, USP8 ↓, E2f, FSH, Vegf, ADRB, ERK1/2, Lh, Ap1, Mapk, Cdk, DUB, NADPH oxidase, pro-infammatory sytokine	51	23 (ADCY4 ↑, CDKN3 ↓, NEK2 ↓, PGK1 ↓, S100A12 ↓, UBLCP1 ↓, DAB2 ↑, NOTCH2 ↓, PNP ↓, TGFA ↓, UCHL5 ↓, VPS50 ↓, DEFB103A/DEFB103b ↓, FANCG ↑, PAIP2 ↓, PPM1D ↓, TMEM126A ↓, USP16 ↓, YES1 ↓, FCHO2 ↓, PBK ↓, TTK ↓, USP8 ↓)	Antimicrobial response, cellular function and maintenance, inflammatory response
2	CLTC ↓, HAT1 ↓, HMGB1 ↓, KRT3 ↓, SLBP ↓, NET1 ↓, PRDM1 ↓, BCL10 ↓, CD58 ↓, DDR2 ↑, NUP54 ↓, SDHD ↓, TNFRSF14 ↑, CEACAM1 ↓, DTL ↓, INPPL1 ↑,ORC3 ↓, SERPING1 ↑, actin,	37	18 (CLTC ↓, HAT1 ↓, HMGB1 ↓, KRT3 ↓, SLBP ↓, NET1 ↓, PRDM1 ↓, BCL10 ↓, CD58 ↓, DDR2 ↑, NUP54 ↓, SDHD ↓, TNFRSF14 ↑,	Cellular movement, hematological system development and

	caspase, IL1, PI3K (complex), Akt, CD3, collagen(s), HISTONE, IgG, IL12 (complex), Tgf beta, histone H3, IgG2a, immunoglobulin, BCR (complex), histone h4, Igm		CEACAM1 ↓, DTL ↓, INPPL1 ↑,ORC3 ↓, SERPING1 ↑)	function, immune cell trafficking
3	NISCH ↑, PUDP ↓, BRIX1 ↓, NM37 ↓, TGM5 ↓, VSIG8 ↓, CLPX ↓, METTL5 ↓, TMEM154 ↓, CRYZ ↓, MICU2 ↓, PSMG1 ↓, SHANK3 ↑, TMEM199 ↓, ACOX2, beta-estradiol, CTNNB1, SLC17A1, SPATA13, TTC17, ACOX3, ESR2, Macf1, RAB5C, SLC38A5, ARHGAP44, HCN3, NXF1, RHBDF1, SLFN11, WDTC1, BAMBI, HNF4A, SMARCA4		14 (NISCH ↑, PUDP ↓, BRIX1 ↓, NM37 ↓, TGM5 ↓, VSIG8 ↓, CLPX ↓, METTL5 ↓, TMEM154 ↓, CRYZ ↓, MICU2 ↓, PSMG1 ↓, SHANK3 ↑, TMEM199 ↓)	Cancer, cell death and survival, organismal injury and abnormalities
4	KIF11 ↓, PCLAF ↓, PLCH2 ↑, SMPDL3A ↓, UQCRH ↓, EIF1AX ↓, NNT ↓, SPRR2B ↓, TTC33 ↓, ZFAND1 ↓, TAF1B ↓, TXNDC9 ↓, ADGRE5, CKMT2, EXPH5, NAB1, SL1, TP53, UQCR10, ADGRL1, CTIF, FAM84B, LCOR, NEIL3, TSPAN15, AGTR1, GPRC5C, LZTS1, PLEKHA3, CELSR3, ELAVL1, hydrogen peroxide, Mpf, NT5DC3, progesterone	22	12 (KIF11 ↓, PCLAF ↓, PLCH2 ↑, SMPDL3A ↓, UQCRH ↓, EIF1AX ↓, NNT ↓, SPRR2B ↓, TTC33 ↓, ZFAND1 ↓, TAF1B ↓, TXNDC9 ↓)	Cell death and survival, cellular compromise, hair and skin development and function
5	AFTPH ↓, RPA3 ↓, TCEAL9 ↓, DST ↓, RAB6A ↓, HTR3A ↓, LXN ↓, RAD51AP1 ↓, SMC2 ↓, IRX3 ↓, OLFML3 ↑, SRP19 ↓, DOK4, EGFR ligand, GPAA1, LIG4, NCAPG2, PAXX, ANKS1B, Egfr-Erbb2, GRID1, LMBR1, NHEJ1, SLFN11, TRMO, C1QL4, EBP, ERBB2, NTHL1, UQCR10, COL17A1, EGFR, FUT8, MAP7D2, RIC3	22	12 (AFTPH ↓, RPA3 ↓, TCEAL9 ↓, DST ↓, RAB6A ↓, HTR3A ↓, LXN ↓, RAD51AP1 ↓, SMC2 ↓, IRX3 ↓, OLFML3 ↑, SRP19 ↓)	Cell cycle, cell morphology, DNA replication, recombination, and repair
6	DEFB103A/DEFB103b ↓, KLF5 ↓, DLGAP5 ↓, KTN1 ↓, PAK1IP1 ↓, ACVRL1 ↑, PDCD10 ↓, C1QTNF5 ↑, KCNK1 ↓, S100A12 ↓, 26s Proteasome, Calmodulin, GJC1, Mmp, P38 MAPK, PELI2, TAC4, acetaldehyde, CGREF1, insulin, neopretin, Pkc(s), TCR, Crisp1/Crisp3, ERK, Jnk, MAP3K13, NEUROD6, RHBDD3, Trbd1, cytokine, focal adhesion kinase, MIP1, NFkB (complex), PDGF BB	17	10 (DEFB103A/DEFB103b ↓, KLF5 ↓, DLGAP5 ↓, KTN1 ↓, PAK1IP1 ↓, ACVRL1 ↑, PDCD10 ↓, C1QTNF5 ↑, KCNK1 ↓, S100A12 ↓)	Cardiovascular system development and function, organismal development, tissue development
7	SNHG7 ↑, FAIM2	2	1 (SNHG7 ↑)	Cell cycle, cell death and survival, tissue morphology

Supplementary Table 9. Top two master regulators. Participating regulators are molecules through which the upstream regulator molecule controls the expression of target molecules in dataset. Depth is the degree of separation between the upstream regulator and the downstream target molecules in dataset (1-3). Target connected regulators is the count of the regulators that interact with at least one target molecule in the dataset.

**Master regulator**	**Molecule type**	**Participating regulators**	**Depth**	**Predicted activation**	**Activation z-score**	***p*-value of overlap**	**Network bias-corrected *p*-**	**Target genes in dataset**	**Causal network**
TFIIH	Complex	ATF4, CCND1, CCNH, CDK7, CDK9, CEBPA, CREB1, EGFR, ESR1, GATA1, IL2, MNAT1, MYOD1, NCOA3, NELFE, NFKBIA, NR5A1, PGR, POU5F1, PPARA, PPARG, PPARGC1A, progesterone, SP1, SRC, STAT1, STAT3, SUB1, tamoxifen, TFIIH	3	Inhibited	-2.611	9.71 E-07	2.30 E-03	ACVRL1, ADCY4, BCL10, C1QTNF5, CD58, CDKN3, CEACAM1, DAB2, DDR2, DLGAP5, DST, DTL, EIF1AX, HTR3A, INPPL1, IRX3, KTN1, LXN, OLFML3, PAIP2, PBK, PGK1, PNP, PRDM1, RAB6A, SMPDL3A, TCEAL9, TGFA, TTK, UCHL5, UQCRH, USP16, YES1	33 (30)
AHR	Ligand-dependent nuclear receptor	AHR, Akt, ARNT, EGFR, ERK, ESR1, IL22, Jnk, MITF, NFKBIA, P38 MAPK, RAF1, RB1, RELA, SMAD2, tanespimycin, ZFP36	2	-	0.192	1.38 E-06	3.40 E-03	C1QTNF5, CDKN3, DAB2, DEFB103A/DEFB103B, DST, FANCG, HAT1, KIF11, KLF5, KTN1, NEK2, NET1, NOTCH2, PAIP2, PBK, PCLAF, PGK1, PNP, PPM1D, PRDM1, RAB6A, RPA3, S100A12, SDHD, TCEAL9, TNFRSF14, TTK	27 (17)
